# Impact of prehypertension on left ventricular mass and QT dispersion in adult black Nigerians

**DOI:** 10.5830/CVJA-2014-010

**Published:** 2014-04

**Authors:** OK Ale, JN Ajuluchukwu, DA Oke, AC Mbakwem

**Affiliations:** Department of Medicine, College of Medicine, University of Lagos/Lagos University Teaching Hospital, Lagos, Nigeria; Department of Medicine, College of Medicine, University of Lagos/Lagos University Teaching Hospital, Lagos, Nigeria; Department of Medicine, College of Medicine, University of Lagos/Lagos University Teaching Hospital, Lagos, Nigeria; Department of Medicine, College of Medicine, University of Lagos/Lagos University Teaching Hospital, Lagos, Nigeria

**Keywords:** prehypertension, left ventricular hypertrophy, left ventricular mass, QT dispersion, adult black Nigerian

## Abstract

**Background:**

Prehypertension has been associated with target-organ damage. This study sought to determine the impact of prehypertension (PHT) on QT dispersion and left ventricular hypertrophy (LVH) in adult black Nigerians.

**Methods:**

One hundred and one subjects with office blood pressure (BP) < 140/90 mmHg were categorised according to their office BP into normotensive (BP < 120/80 mmHg, *n* = 57) and prehypertensive (BP 120–139/80–89 mmHg, *n* = 44) groups. Echocardiography and electrocardiography (ECG) were performed on the subjects.

**Results:**

Thirty-four males aged 53.65 ± 16.33 years and 67 females aged 52.42 ± 12.00 years were studied. The mean QT interval dispersion (QTd) of the normotensive (38.96 ± 11.06 ms) and prehypertensive (38.41 ± 11.81 ms) groups were similar (*p* = 0.81). Prehypertensive subjects had higher left ventricular mass (LVM) (165.75 ± 33.21 vs 144.54 ± 35.55 g, *p* = 0.024), left ventricular mass index 1 (LVMI-1) (91.65 ± 16.84 vs 80.45 ± 18.65 g/m^2^, *p* = 0.021) and left ventricular mass index 2 (LVMI-2) (54.96 ± 10.84 vs 47.51 ± 12.00 g/m^2.7^, *p* = 0.017). QTd was independent of echocardiographic and electrocardiographic LVH (*p* > 0.05).

**Conclusion:**

Compared with normotension, prehypertension is associated with higher LVM but similar QTd. This suggests that structural remodelling precedes electrical remodelling in prehypertension.

## Abstract

The heterogeneity of individuals with blood pressure (BP) < 140/90 mmHg in terms of cardiovascular (CV) risk was reported as early as 1939 by Robinson and Brucer.^1^ BP in the range of 120–139/80–89 mmHg (labelled then as prehypertension) was observed to be associated with high risk of progression to hypertension (HT) and cardiovascular disease (CVD) later in life when compared with BP < 120/80 mm Hg.^1^

The term prehypertension was adopted in May 2003 by the Seventh Report of the Joint National Committee on Prevention, Detection, Evaluation and Treatment of High blood Pressure (JNC-7) to describe BP range of 120–139/80–89 mmHg.^2^ The resuscitation of this terminology/concept in JNC-7 was a sequel to the documentation of a higher morbidity in individuals with prehypertension in landmark publications.^3^-^5^ Prehypertension (PHT) was defined in JNC-7 not only to emphasise the excess risk associated with BP in this range, but also to focus increased clinical and public health attention on prevention.^2^,^6^,^7^

Prevalence rates of PHT among adults in the United States, Ghana and northern Nigeria have been reported to be 31, 40 and 58.7%, respectively.^7^-^9^ In most studies, including the ones above, PHT was more prevalent than hypertension.^7^-^9^ Though PHT is associated with increased risk of major CV events independently of other CV risk factors,^10^ most individuals (90%) with PHT have at least one cardiovascular risk factor such as dyslipidaemia, abdominal obesity, hyperinsulinaemia, impaired fasting glucose levels, insulin resistance, a prothrombotic state, tobacco use, endothelial dysfunction, and impaired vascular distensibility.^6^,^7^,^9^,^10^

QT interval dispersion (QTd) (the difference between the longest and the shortest QT intervals on a surface ECG), when excessive, is associated with increased risk of cardiovascular morbidity and mortality in population studies, and many clinical conditions, including hypertension.^11^,^12^ This has been related to ventricular electrical instability, providing the necessary substrate for lethal ventricular arrhythmias.^12^,^13^ Greater QTd and left ventricular mass have been demonstrated in hypertensive individuals compared with normal individuals.^11^,^13^,^14^

Considering the well-established, linear relationship between BP and the risk of cardiovascular events, the CV risk associated with PHT is intermediate between normotension and hypertension.^2^,^03^ Hence, electrocardiographic and echocardiographic indices of target-organ damage in PHT may also be intermediate between normotension and hypertension. The aims of this study were: (1) to compare the QTd and indices of left ventricular hypertrophy in adult black normal and prehypertensive subjects, and (2) to evaluate the relationship of QTd with electrocardiographic and echocardiographic indices in these subjects.

## Methods

One hundred and one consecutive, apparently healthy black Nigerian students, staff and retirees of the Lagos University Teaching Hospital and the College of Medicine, University of Lagos, aged between 26 and 86 years were recruited. They fulfilled the following criteria: age ≥ 18 years, no history of heart disease, including hypertension or other conditions known to affect QT interval and QTd (e.g. diabetes mellitus, dysautonomia), normal cardiac physical examination and fasting blood sugar level < 7 mmol/l. None of the subjects was on treatment with drugs known to affect QT interval (e.g. statins, macrolide antibiotics, halofantrine, amiodarone).^15^

Exclusion criteria were the presence of sustained non-sinus rhythm, intraventricular conduction defects and electrocardiograms in which the end of the T waves could not be reliably determined and/or QT interval from less than eight leads could be analysed.^11^ Subjects with suboptimal echo windows were also excluded from echocardiographic examination.

Ethical clearance was obtained from the ethics and research committee of the Lagos University Teaching Hospital. The study was conducted to conform to the ethical tenets developed by the World Medical Association, as espoused in the Declaration of Helsinki. All subjects provided informed consent.

All subjects were classified by office BP as either normotensive: normal BP (< 120/80 mmHg) (*n* = 57) or prehypertensive: prehypertensive BP (120–139/80–89 mmHg) (*n* = 44), according to the JNC-7 recommendations.^2^

A detailed medical history was obtained, physical examination was performed and anthropometric variables of height and weight were obtained. Trained personnel obtained measures of height and weight using a calibrated stadiometer and a weighing scale. Each subject’s height was measured without shoes, in the standing position, heels together, toes apart at a 45° angle and the head in the Frankfort horizontal plane. Weight was measured with the subject lightly clothed and without shoes. Body mass index (BMI) was calculated according to the formula:^16^ BMI = body mass (kg)/body height^2^ (m^2^). BMI < 25 kg/m^2^, 25 to < 30 kg/m^2^, and ≥ 30 kg/m^2^ were classified as normal, overweight and obese, respectively.^16^

Resting blood pressure was measured three times for each subject with a standard mercury sphygmomanometer on the right arm in a sitting position following a minimum of five minutes’ rest by a physician. Phases I and V Korotkoff sounds were used to determine systolic and diastolic BP measurements. The mean of the last two measurements was used in the analysis.

## Electrocardiography

All subjects had a resting simultaneous 12-lead electrocardiogram (ECG) using an Esaote P80 Power electrocardiograph machine. At a paper speed of 25 mm/s with the machine control set at standard response, a standard lead II rhythm strip of 13–16 complexes and a minimum of three cardiac cycles per lead were recorded. All electrocardiograms were analysed by a single observer blinded to the clinical data.

QT and the preceding RR intervals were assessed manually with callipers and mean values were determined in three consecutive cycles. QT intervals were measured in all possible leads from the beginning of the QRS complex to the point of T wave offset, i.e. the point of the return of the T wave to the isoelectric line.^11^ In the presence of the U wave interrupting the T wave, the nadir between the T and U waves was used to define the point of T wave offset.

QT dispersion (QTd) in milliseconds was defined as the difference between the shortest (QT_min_) and longest (QT_max_) mean QT interval in each electrocardiogram.^11^ The QT interval was measured from the lead with the longest interval and was corrected for subjects’ heart rate using Bazett’s formula: 17

$QTc=\frac{QT_{0}}{\sqrt{RR}}$

where QTc is the corrected QT interval, QTo is the observed QT interval in milliseconds, RR is the RR interval in milliseconds. QTc ≤ 440 ms and QTd = 30–60 ms were considered normal.^18^-^20^

ECG LVH was determined using the Araoye’s criteria^21^,^22^ for LVH in blacks [i.e. (1) SV_2_ + RV_6_ > 4.0 mV in males ≥ 30 years, SV_2_ + RV_6_ > 5.0 mV in males aged 15–29 years and SV_2_ + RV_6_ > 3.5 mV in females; (2) flat or inverted T waves in V5 or V_6_; (c) R1 amplitude > 1.2 mV. ECG LVH is diagnosed when any of the criteria is positive] and the Sokolow–Lyon voltage criteria.23 Araoye’s criteria has been shown to correlate well with echocardiographic LVH in Nigerians.^24^

## Echocardiography

Transthoracic echocardiography was performed on the first 60 consecutive subjects using a Hewlett Packard Sonos 2000 machine. Using the American Society of Echocardiography (ASE) recommendations,^25^ the following measurements were obtained: left ventricular internal diameter in diastole (LVIDd), left ventricular internal diameter in systole (LVIDs), interventricular septal thickness in diastole (IVSTd) and left ventricular posterior wall thickness in diastole (PWTd).

Left ventricular mass (LVM) was derived using the ASE formula:^25^

Estimated LVM (g) = 0.80 [1.04 (LVIDd + PWTd + IVSTd)^3^ – LVIDd)^3^] + 0.6 g

Left ventricular mass index was determined using two different methods. Left ventricular mass index 1 (LVMI-1) was calculated as the ratio of LVM to body surface area (g/m^2^).^25^ Subjects were was considered to have LVH if LVMI-1 was more than 134 g/m^2^ for men and more than 110 g/m^2^ for women.^26^

The second LVMI, i.e. LVMI-2 was derived by indexing LVM to height using the formula:^27^

LVMI-2 (g/m^2.7^) = LVM/height^2.7^.

However only LVMI-1 was used for the determination of the presence or absence of LVH in the subjects.

## Statistical analysis

The SPSS 17.0 statistical software was used for data analysis. The data obtained were expressed as means and proportions. Statistical significance of variables was tested using the chi-square and Fisher’s exact test for categorical variables and the Student’s *t*-test for continuous variables. Analysis of variance was used to assess intra-observer variability of height and weight measures of the first 35 subjects.

The strengths of the relationship between QTd and selected continuous variables were assessed with the Pearson’s correlation coefficient. Variables that demonstrated significant relationship to QT dispersion i.e. LVM, LVMI and LVMI-2 in the prehypertensive group were entered as independent variables into a standard (simultaneous) multiple regression model with QTd as the dependent variable. All tests were two-sided and values were considered statistically significant if *p* < 0.05.

## Results

A total of 101 subjects aged between 26 and 86 years were enrolled into the study. The age of the normotensive (*n* = 57) and prehypertensive group (*n* = 44) of subjects ranged from 27–86 and 26–78 years, respectively. The ICC for the intra-observer assessment of the measures of height and weight are 0.97 and 0.96, respectively.

Nineteen (43%) of the prehypertensive subjects had both systolic (SBP) and diastolic blood pressure (DBP) within the prehypertensive range. This subpopulation of the prehypertensives with a mean age of 53.37 ± 10.96 years consisted of four male and 15 female subjects. Their mean QTd of 39.21 ± 13.46 ms was similar to QTd of the rest of the cohort (*p* > 0.05).

The clinical characteristics of the study group are presented in Table 1. There were no differences in the age, gender and anthropometric measurements of the two groups. The prehypertensive group however had significantly higher BP indices.

**Table 1:** Clinical characteristics of the study population

*Characteristic*	*Total population n = 101 Mean ± 2SD n (%)*	*Normotensive group n = 57 Mean ± 2SD n (%)*	*Prehypertensive group n = 44 Mean ± 2SD n (%)*	p*-value*
Age	59.96 ± 13.54	50.74 ± 13.89	55.84 ± 12.65	0.06
Gender				0.94
Male	34 (33.7)	19 (33.3)	15 (34.1)	
Female	67 (66.3)	38 (66.7)	29 (65.9)	
Weight (kg)	69.10 ± 13.10	68.02 ± 12.32	70.50 ± 14.05	0.20
Height (m)	1.63 ± 0.08	1.63 ± 0.07	1.62 ± 0.10	0.44
BMI (kg/m^2^)	26.17 ± 4.72	25.61 ± 4.63	26.89 ± 4.78	0.18
BMI class				0.35
Normal	45 (44.6)	29 (50.9)	16 (36.4)	
Overweight	35 (35.6)	18 (31.6)	18 (40.9)	
Obese	20 (19.8)	10 (17.5)	10 (22.7)	
SBP (mmHg)	118.27 ± 19.50	112.39 ± 6.24	125.89 ± 7.34	< 0.001
DBP (mmHg)	73.70 ± 7.43	71.05 ± 6.60	77.14 ± 7.10	< 0.001
MAP (mmHg)	88.56 ± 6.98	84.83 ± 5.61	93.39 ± 5.48	< 0.001
PP (mmHg)	44.56 ± 9.04	41.33 ± 6.86	48.75 ± 9.83	< 0.001

BMI, body mass index; SBP, systolic blood pressure; DBP, diastolic blood pressure; MAP, mean arterial blood pressure; PP, pulse pressure.

The electrocardiographic measures of the two groups are presented in Table 2. The heart rate, QTc, QTd and ECG LVH status of the two groups were similar.

**Table 2:** ECG measurements according to BP group.

*Characteristic*	*Total population n = 101 Mean ± 2SD n (%)*	*Normotensive group n = 57 Mean ± 2SD n (%)*	*Prehypertensive group n = 44 Mean ± 2SD n (%)*	p*-value*
Heart rate (beats/min)	73.35 ± 10.94	72.11 ± 9.53	74.95 ± 12.47	0.38
QRS (ms)	79.96 ± 3.51	79.54 ± 3.46	80.50 ± 3.53	0.18
QTd (ms)	38.72 ± 11.34	38.96 ± 11.06	38.41 ± 11.81	0.81
QTc (ms)	417.92 ± 23.63	415.58 ± 23.25	420.95 ± 24.04	0.26
ECG LVH				
Sokolow–Lyon	8 (7.9)	4 (7.0)	4 (9.1)	0.73
Araoye’s code	10 (9.9)	4 (7.0)	6 (13.6)	0.33

QTc, corrected QT interval; QTd, QT dispersion, QRS, QRS duration, LVH, left ventricular hypertrophy.

Table 3 shows the echocardiographic measurements of the groups. The prehypertensive group had significantly higher IVSTd, LVM, LVMI-1 and LVMI-2 values.

**Table 3:** Echocardiographic measurements according to BP groups

*Characteristic*	*Total population n = 60 Mean ± 2SD n (%)*	*Normotensive group n = 36 Mean ± 2SD n (%)*	*Prehypertensive group n = 24 Mean ± 2SD n (%)*	p*-value*
LVIDd (cm)	4.52 ± 0.44	4.50 ± 0.41	4.55 ± 0.49	0.64
LVIDs (cm)	2.90 ± 0.43	2.93 ± 0.41	2.84 ± 0.46	0.43
IVSTd (cm)	1.05 ± 0.21	1.00 ± 0.20	1.15 ± 0.21	0.01
LVPWd (cm)	0.88 ± 0.14	0.88 ± 0.15	0.91 ± 0.13	0.29
RWT (cm)	0.40 ± 0.09	0.39 ± 0.09	0.41 ± 0.09	0.57
LVM (g)	153.03 ± 35.91	144.54 ± 35.55	165.75 ± 33.21	0.024
LVMI-1(g/m^2^)	84.93 ± 18.64	80.45 ± 18.65	91.65 ± 16.84	0.021
LVMI-2 (g/m^2.7^)	50.49 ± 12.02	47.51 ± 12.00	54.96 ± 10.84	0.017
EF (%)	72.72 ± 8.73	71.44 ± 8.57	74.65 ± 8.80	0.17
Echo LVH	2(3.33)	1(1.67)	1(1.67)	1.00

LVM, left ventricular mass; LVMI, left ventricular mass index; LVH, left ventricular hypertrophy; LVIDd, left ventricular internal diameter in diastole; LVIDs, left ventricular internal diameter in systole; IVSTd, interventricular septal thickness in diastole; PWTd, left ventricular posterior wall thickness in diastole.

The relationships of QTd to LVH determined by ECG and echocardiography are presented in Table 4. QTd was independent of LVH status, not only in the whole cohort, but also in the two groups.

**Table 4:** Relationship of QTd to LVH status.

*LVH classification*	*LVH status*	*Total population QTd (ms)*	p*-value*	*Normotensive group QTd (ms)*	p*-value*	*Prehypertensive group QTd (ms)*	p*-value*
Sokolow–Lyon	No LVH	38.77 ± 11.31	0.88	39.11 ± 11.10	0.72	38.33 ± 11.72	0.88
	LVH	38.13 ± 12.39		37.00 ± 11.94		39.25 ± 14.57	
Araoye	No LVH	38.78 ± 10.78	0.88	39.36 ± 10.89	0.33	39.97 ± 10.67	0.54
	LVH	38.20 ± 16.44		33.75 ± 13.77		41.17 ± 18.61	
LVMI	No LVH	39.72 ± 10.24	0.97	40.60 ± 10.70	0.96	38.39 ± 9.58	0.87
	LVH	40.00 ± 0.00		40.00 ± 0.00		40.00 ± 0.00	

QTd, QT dispersion; LVH, left ventricular hypertrophy; LVMI, left ventricular mass index.

QTd correlated significantly with LVM (*r* = 0.58, *p* = 0.003), LVMI (*r* = 0.55, *p* = 0.006) and LVMI-2 (*r* = 0.49, *p* = 0.016) in the prehypertensive subjects. Several other variables were tested in both groups but did not correlate with QTd (*p* > 0.05): age, weight, height, BMI, SBP, DBP, pulse pressure (PP), heart rate, QTc, LVIDd, LVIDs, IVSTd, LVPWd, RWT and ejection fraction (EF). None of the independent variables (LVM, LVMI and LVMI-2 in the prehypertensive group) in the simultaneous regression model with QTd as the dependent variable made statistically significant contributions to the equation (computed *R*^2^ = 34%, adjusted *R*^2^ = 24%). The beta-values and the levels of significance were 0.50 and 0.31 for LVM, 0.04 and 0.96 for LVMI and 0.14 and 0.96 for LVMI-2.

## Discussion

PHT is an intermediate stage between normal BP and hypertension. The mechanisms of excess CV risk/end-organ damage associated with PHT are presumed to be the same as that of hypertension. This suggests that CV risk and end-organ damage in PHT, together with their surrogates such as QTd and LVH, are also intermediate.

Increased QTd is a marker of increased myocardial electrical instability, which has been associated with hypertension.^11^,^13^,^14^ This study showed similar QTd values in normal and PHT subjects. This is at variance with the report by Dogru *et al*.^28^ of higher QT dispersion in prehypertensives when compared with normotensive individuals.

Racial differences may contribute to this discrepancy. Studies have suggested significant differences in the cardiac structure and function of subjects of African descent compared with non-negroid subjects.^29^,^30^ This was further demonstrated in a study by Zhu *et al.* of white and black PHT subjects,^31^ which suggested that cardiovascular characteristics of prehypertension appear to be race dependent.

The subjects in our study may have had lower BP values, i.e. closer to normotension than those in the above study, with a consequent blunting of the expected difference in the QTd values of the subjects with normal BP and PHT. Data have suggested a wide variation in the QTd of normal individuals (BP < 140/90 mmHg), hence it has been difficult to define what constitutes a normal QTd.^11^ This variation may reduce the ability of QTd to discriminate between prehypertension and normotension, with a consequent similarity observed in the QTd values of prehypertensive and normotensive subjects in this study.

LVH, a compensatory mechanism for ventricular overload, is an independent risk factor for CV morbidity and mortality in normotensive and hypertensive individuals.^32^,^33^ The present study showed higher indices of LVH, i.e. IVSTd, LVM and LVMI in the PHT subjects. Manios *et al.*^34^ and Drukteinis *et al.*^35^ documented similar findings of higher LVM in prehypertensives than in normotensives, even after adjusting for co-variates. Conversely, Zhu *et al.*^31^ reported similar LVM values in normotensive and hypertensive subjects. Differences in study population and the methodology of BP measurement (use of ambulatory or office BP and different protocols) may have accounted for this variation.

The normotensive and prehypertensive groups in the present study were however similar in terms of ECG LVH status. This may be attributed to the low sensitivity of ECG criteria in detecting LVH, therefore limiting their ability to measure milder changes in LVM expected in prehypertension.^36^,^37^ The report of Ang and Lang,^38^ that the sensitivities of ECG LVH criteria are substantially lower when tested in the general population than in a high-risk population, such as hypertensive patients, gives further credence to this view. Both ECG LVH and echocardiographic LVH as prognostic factors for CVD may reflect different pathological processes and thereby influence prognosis in different ways.^38^

The demonstration of similar QTd in normotensive and prehypertensive subjects, together with a concomitantly higher LVM in PHT seen in our data suggests that left ventricular structural remodelling precedes electrical remodelling in a continuum of cardiovascular changes induced by increasing BP in prehypertension. This probably confers a higher sensitivity to LVM measurement over ECG parameters, such as QTd and LVH measurement in the detection of prehypertensive changes in the myocardium.

The relatively small study population and the recording of the ECG at a speed of 25 mm/s, which is the usual speed of ECG recordings in clinical practice, were limitations in this study. QT interval measurements are more reproducible at faster paper speed recordings.^11^ Moreover, the cross-sectional design of this study precludes the establishment of a cause–effect relationship between prehypertension and increased LVM. This relationship, including the likelihood of reverse causality between LVM and prehypertension, will be better addressed by a prospective study. However, the findings of this study can serve as a basis for further studies on the effects of prehypertension in adult black Nigerians.

## Conclusions

The findings of similar ECG parameters (QTd and ECG LVH) in prehypertensive and normotensive subjects suggest a limitation in the usefulness of ECG for CV risk stratification in prehypertension. Conversely, echocardiography may be a good screening tool for the detection of prehypertensive changes in the heart. However, this may not be feasible in resource-poor countries such as Nigeria.
